# Mortality of Philadelphia for January, February and March, 1855; Collated from the Record at the Health Office

**Published:** 1855-05

**Authors:** Wilson Jewell


*Mortality of Philadelphia for January, February and March,* 1855*; collated from the Record at the Health Office.* By Wilson Jewell, M.D.

The number of deaths in our city for the 1st quarter of the present year, 1855, embracing the months of January, February and March, amount to 2,526, a fraction less than for the same period of last year. This number furnishes an average of 27½ deaths per day for 92 days.†

The mortality ascribed to diseases alone, as shown by the record at the Health Office, is 2076. Hence, 450 of the deaths are charged to Still-Born, External Causes, Old Age, Debility and Unknown.

From the male sex the deaths reached 1318; while from the female sex they were only 1208; showing an excess in the former, equivalent to 4·35 per cent.

In the deaths from External Causes, the excess amongst males amounts to 37·25 per cent.; attributable, no doubt, to their greater exposure than the other sex.

From diseases of the Nervous System, the excess of deaths is also on the side of the males, and is equal to 14·28 per cent.

It will be observed, that 1030 of the deaths, exclusive of Still-Born, or 43·02 per cent., took place within the 5th year of life; and that 1240, or 51·79 per cent., occurred within the 20th year.

*About one-seventh of the price of the sulphate of quinia.

† The quarter includes the two last days of December, 1854.

As in former years so in this, the infant mortality bears a far less per centage to the whole number of deaths during the winter than in the summer and autumnal months.

The prevalent diseases during the quarter have been those of the phlegmasia, involving principally the respiratory organs, which constitute 35.79 per cent. of all the deaths from accredited diseases, and also those of the nervous system, which amount to 22·25 per cent.

Under these two classifications of deaths, we note that Consumption of the Lungs furnish 377, or 18·15 per cent. of the deaths from diseases alone. Inflammation of the Lungs, 174; Inflammation of the Bronchiæ, 89; Convulsions, 131; Dropsy of the Brain, 78; Inflammation of the Brain, 73; Congestion of the Brain, 44.

Among some of the exanthemata, the deaths have been lighter than last year in similar months. Those from Scarlet Fever have fallen off 48·61 per cent., and from measles one-half. The deaths from Small Pox and Varioloid, on the other hand, have increased 66·03 per cent.

The deaths from Croup have been 5 per cent. less than for the same quarter of last year.

The deaths from Old Age number 55, equal to one in 43 of all the deaths, exclusive of Still-born, or 1·32 per cent.

From the Blockley Almshouse there were 234 deaths, or 9·24 per cent. of the whole mortality for the quarter. The black population contributed 140, or 5·54 per cent. to the bill of mortality.

TABLE NO. 1.

*Deaths for the first quarter of 1855 classified.*

| | | | | Male. | | | Female. | | | |
|---|---|---|---|---|---|---|---|---|---|---|
| | Jan. | Feb. | Mar. | J. | F. | M. | J. | F. | M. | Total. |
| 1 *Endemic & Contagious diseases* Zymotic or Epidemic | 120 | 135 | 134 | 69 | 61 | 72 | 51 | 74 | 62 | 389 |
| 2 *Uncertain or general seat,* Sporadic diseases | 112 | 108 | 103 | 53 | 51 | 49 | 59 | 57 | 54 | 323 |
| 3 Nervous system | 150 | 140 | 172 | 77 | 74 | 113 | 73 | 66 | 59 | 462 |
| 4 Organs of Respiration | 252 | 220 | 271 | 131 | 117 | 129 | 121 | 103 | 142 | 743 |
| 5 " Circulation | 30 | 20 | 27 | 14 | 13 | 18 | 16 | 7 | 9 | 77 |
| 6 Digestive organs | 46 | 41 | 51 | 23 | 19 | 21 | 23 | 22 | 30 | 138 |
| 7 Urinary " | 4 | 1 | 2 | 2 | 1 | 2 | 2 | | | 7 |
| 8 Organs of Generation | 11 | 9 | 8 | | | | 11 | 9 | 8 | 28 |
| 9 " Locomotion | 6 | 1 | 3 | 1 | | 1 | 5 | 4 | 2 | 10 |
| 10 Integumentary system | 3 | | | 1 | | | 2 | | | 3 |
| 11 Old age | 14 | 15 | 26 | 6 | 6 | 13 | 8 | 9 | 13 | 55 |
| 12 External causes | 44 | 24 | 34 | 32 | 17 | 21 | 12 | 7 | 13 | 102 |
| Still Born | 52 | 36 | 44 | 31 | 16 | 30 | 21 | 20 | 14 | 132 |
| Unknown | 22 | 18 | 17 | 10 | 12 | 12 | 12 | 6 | 5 | 57 |
| | 866 | 768 | 892 | 450 | 387 | 481 | 416 | 381 | 411 | 2526 |

### TABLE NO. 2.

### 1. *Endemic and Contagious Diseases—Zymotic or Epidemic.*

| | Male. | Female. | Under 1 yr. | 1 to 2. | 2 to 5. | 5 to 10. | 10 to 15. | 15 to 20. | 20 to 30. | 30 to 40. | 40 to 50. | 50 to 60. | 60 to 70. | 70 to 80. | 80 to 90. | 90 to 100. | 100 to 110. | Total. |
|---|---|---|---|---|---|---|---|---|---|---|---|---|---|---|---|---|---|---|
| Cholera Asphyxia | 1 | 1 | | | | | | | 2 | | | | | | | | | 2 |
| " infantum | 3 | 2 | 2 | 1 | 2 | | | | | | | | | | | | | 5 |
| Croup | 44 | 45 | 19 | 32 | 27 | 11 | | | | | | | | | | | | 89 |
| Diarrhœa | 14 | 12 | 9 | 5 | 2 | | | | 2 | 2 | | | 5 | 1 | | | | 26 |
| Dysentery | 21 | 7 | 4 | 4 | 3 | 2 | 1 | 1 | 5 | 3 | 3 | | 1 | 1 | | | | 28 |
| Erysipelas | 15 | 12 | 10 | 3 | | 1 | | | 3 | 2 | 1 | 3 | 1 | 2 | 1 | | | 27 |
| Fever, | 3 | 3 | 3 | | 1 | 1 | 1 | | | | | | | | | | | 6 |
| " Bilious | 2 | | | | | 1 | | | | | | 1 | | | | | | 2 |
| " Continued | 1 | | | | 1 | | | | | | | | | | | | | 1 |
| " Intermit. | 1 | | | 1 | | | | | | | | | | | | | | 1 |
| " Puerperal | | 15 | | | | | | 2 | 8 | 5 | | | | | | | | 15 |
| " Remittent | 5 | 4 | 1 | 2 | 2 | 1 | 1 | | 1 | | | | | 1 | | | | 9 |
| " Scarlet | 17 | 19 | 4 | 2 | 15 | 10 | 4 | | 1 | | | | | | | | | 36 |
| " Typhoid | 31 | 24 | | 2 | 1 | 1 | 5 | 10 | 20 | 8 | 2 | 4 | | | 2 | | | 55 |
| " Typhus | 7 | 6 | | | | 3 | 1 | 1 | 3 | 3 | | 1 | 1 | | | | | 13 |
| Hooping Cough | 5 | 13 | 5 | 9 | 4 | | | | | | | | | | | | | 18 |
| Measles | 7 | 4 | 5 | 4 | 1 | 1 | | | | | | | | | | | | 11 |
| Small Pox | 23 | 18 | 13 | 4 | 11 | 6 | | 3 | 2 | 1 | 1 | | | | | | | 41 |
| Syphilis | | 1 | | | | | | | | | 1 | | | | | | | 1 |
| Varioloid | 2 | 1 | 1 | 1 | 1 | | | | | | | | | | | | | 3 |
| | 202 | 186 | 76 | 70 | 71 | 38 | 13 | 17 | 47 | 24 | 8 | 9 | 8 | 5 | 3 | | | 389 |

### 2. *Uncertain or General Seat—Sporadic Diseases.*

| | Male. | Female. | Under 1 yr. | 1 to 2. | 2 to 5. | 5 to 10. | 10 to 15. | 15 to 20. | 20 to 30. | 30 to 40. | 40 to 50. | 50 to 60. | 60 to 70. | 70 to 80. | 80 to 90. | 90 to 100. | 100 to 110. | Total. |
|---|---|---|---|---|---|---|---|---|---|---|---|---|---|---|---|---|---|---|
| Abscess | | 1 | | | 1 | | | | | | | | | | | | | 1 |
| " Psoas | 1 | | | | | | | 1 | | | | | | | | | | 1 |
| Cancer | 1 | | | | | | | | 1 | | | | | | | | | 1 |
| " Breast | | 1 | | | | | | | | | | | | 1 | | | | 1 |
| Carcinoma | | 1 | | | | | | | | | | 1 | | | | | | 1 |
| Cyanosis | 4 | 1 | 4 | 1 | | | | | | | | | | | | | | 5 |
| Debility | 49 | 55 | 44 | 2 | 2 | | | | 4 | 6 | 3 | 8 | 14 | 16 | 1 | 4 | | 104 |
| Dropsy | 17 | 25 | 1 | 1 | 3 | | 3 | 1 | 2 | 8 | 8 | 3 | 9 | 2 | 1 | | | 42 |
| Fungus Antrum | | 1 | | | | | | | | | | 1 | | | | | | 1 |
| " Hematodes | 1 | | | | | | | | | | | | 1 | | | | | 1 |
| Gangrene | 1 | 2 | | 1 | | | | | 1 | | | | | 1 | | | | 3 |
| " of Arm | 1 | | | | | | | | 1 | | | | | | | | | 1 |
| Gout | | 2 | | | | | | | | | | | 2 | | | | | 2 |
| Hemorrhage | 3 | 4 | 3 | 1 | | | | | | 1 | 1 | | | 1 | | | | 7 |
| Inanition | 11 | 12 | 18 | 2 | | | | | | 1 | 1 | | 1 | | | | | 23 |
| Inflammation | 1 | 1 | | 2 | | | | | | | | | | | | | | 2 |
| " throat | 1 | 2 | | | | | | | | 2 | 1 | | | | | | | 3 |
| " parotid glands | | 1 | 1 | | | | | | | | | | | | | | | 1 |
| Malformation | 7 | 10 | 15 | 2 | | | | | | | | | | | | | | 17 |
| Marasmus | 37 | 45 | 43 | 18 | 10 | 1 | | 1 | 1 | 2 | | 4 | 1 | 1 | | | | 82 |
| Mortification | 1 | 1 | 1 | | 1 | | | | | | | | | | | | | 2 |
| Schirrhus | 1 | | | | | | | | | | | | 1 | | | | | 1 |
| Scrofula | 8 | 3 | 2 | 3 | 2 | 1 | 1 | | 1 | | 1 | | | | | | | 11 |
| Tabes Mesenterica | 4 | 2 | 2 | 1 | | | | 1 | | | | 1 | 1 | | | | | 6 |
| Ulceration of Throat | 4 | | 2 | 1 | | | | | | 1 | | | | | | | | 4 |
| | 153 | 170 | 136 | 35 | 19 | 2 | 4 | 4 | 11 | 21 | 15 | 18 | 30 | 22 | 2 | 4 | | 323 |

### 3. *Nervous Diseases.*

| | Male. | Female. | Under 1 yr. | 1 to 2. | 2 to 5. | 5 to 10. | 10 to 15. | 15 to 20. | 20 to 30. | 30 to 40. | 40 to 50. | 50 to 60. | 60 to 70. | 70 to 80. | 80 to 90. | 90 to 100. | 100 to 110. | Total. |
|---|---|---|---|---|---|---|---|---|---|---|---|---|---|---|---|---|---|---|
| Abscess of Brain | 1 | | | | 1 | | | | | | | | | | | | | 1 |
| Apoplexy | 14 | 12 | 1 | | | | | | | 2 | 3 | 7 | 8 | 2 | | 3 | | 26 |
| Coma | | 1 | | | | | 1 | | | | | | | | | | | 1 |
| Congestion of Brain | 27 | 17 | 9 | 3 | 5 | 8 | | | 4 | 2 | 2 | | 6 | 2 | 3 | | | 44 |
| Concussion " | 2 | | | | | | | | | 1 | 1 | | | | | | | 2 |
| Convulsions | 68 | 63 | 80 | 27 | 17 | 1 | | 2 | 1 | 3 | | | | | | | | 131 |
| Disease of Brain | 18 | 9 | 6 | 8 | 6 | | 2 | | 1 | 2 | | 1 | 1 | | | | | 27 |
| Dropsy " | 50 | 28 | 35 | 19 | 15 | 5 | 2 | | | | 1 | | 1 | | | | | 78 |
| Effusion " | 4 | 13 | 3 | 5 | 2 | 2 | | 2 | 1 | 1 | | | | | 1 | | | 17 |
| Epilepsy | 2 | 3 | 1 | | 1 | | | 1 | 1 | | | | | 1 | | | | 5 |
| Fever, Brain | 1 | 1 | 1 | | 1 | | | | | | | | | | | | | 2 |
| Inflam. " | 42 | 30 | 15 | 11 | 14 | 9 | | 1 | 7 | 5 | 3 | 3 | 3 | 1 | | | | 72 |
| Irritation, " | | 1 | | | 1 | | | | | | | | | | | | | 1 |
| Mania | 3 | 1 | | | | | | | | 1 | 2 | 1 | | | | | | 4 |
| " a Potu | 8 | 3 | | | | | | | 6 | 4 | 1 | | | | | | | 11 |
| " Puerperal | | 2 | | | | | | | 2 | | | | | | | | | 2 |
| Palsy | 20 | 10 | 1 | | | | | | | 2 | 2 | 6 | 7 | 7 | 5 | | | 30 |
| " of Brain | | 2 | 1 | | 1 | | | | | | | | | | | | | 2 |
| Softening " | 3 | 1 | | | 1 | | | | 1 | | | | 2 | | | | | 4 |
| Tetanus | 1 | 1 | | | | | | | | | 1 | 1 | | | | | | 2 |
| | 264 | 198 | 153 | 73 | 65 | 25 | 5 | 6 | 24 | 23 | 16 | 19 | 28 | 13 | 9 | 3 | | 462 |

### 4. *Organs of Respiration.*

| | Male. | Female. | Under 1 yr. | 1 to 2. | 2 to 5. | 5 to 10. | 10 to 15. | 15 to 20. | 20 to 30. | 30 to 40. | 40 to 50. | 50 to 60. | 60 to 70. | 70 to 80. | 80 to 90. | 90 to 100. | 100 to 110. | Total. |
|---|---|---|---|---|---|---|---|---|---|---|---|---|---|---|---|---|---|---|
| Abscess of Lungs | | 1 | | | | | | | | 1 | | | | | | | | 1 |
| Asthma | 2 | 3 | | | | | | | | | | 3 | | 2 | | | | 5 |
| Congestion of Lungs | 22 | 15 | 10 | 4 | 2 | 3 | | 2 | 1 | 4 | | 3 | 6 | 2 | | | | 37 |
| Consumption " | 191 | 186 | 7 | 8 | 8 | 5 | 6 | 28 | 152 | 88 | 41 | 26 | 16 | 7 | 4 | 1 | | 377 |
| Disease of " | 5 | 7 | 3 | 1 | | 2 | | | 2 | | | 1 | 1 | 2 | | | | 12 |
| Dropsy of Chest | 7 | 8 | 1 | | | | 1 | 1 | 3 | 2 | 1 | 1 | 3 | 2 | | | | 15 |
| Effusion of Lungs | 1 | 2 | 2 | | | | | | 1 | | | | | | | | | 3 |
| Hemorrhage of Lungs | 1 | 2 | | | 1 | | | | | | | 1 | 1 | | | | | 3 |
| Hectic Fever | | 1 | | | | | | | 1 | | | | | | | | | 1 |
| Inflamm'n of Bronchiæ | 38 | 51 | 46 | 11 | 9 | | | | 3 | 2 | 2 | 3 | 3 | 6 | 4 | | | 89 |
| " Chest | 3 | 4 | 4 | 1 | 2 | | | | | | | | | | | | | 7 |
| " Larynx | | 2 | 1 | | 1 | | | | | | | | | | | | | 2 |
| " Lungs | 97 | 77 | 53 | 22 | 18 | 4 | 1 | 1 | 13 | 13 | 13 | 8 | 10 | 13 | 3 | 2 | | 174 |
| " Pleura | 6 | 4 | | | | | | | 3 | | 3 | | 3 | 1 | | | | 10 |
| " Tonsils | 1 | | | | 1 | | | | | | | | | | | | | 1 |
| Tuberculosis | 3 | 3 | | 1 | 1 | | | 1 | 1 | 1 | | 1 | | | | | | 6 |
| | 377 | 366 | 127 | 48 | 43 | 14 | 8 | 33 | 160 | 111 | 60 | 47 | 43 | 35 | 11 | 3 | | 743 |

5. *Organs of Circulation.*

| | Male. | Female. | Under 1 yr. | 1 to 2. | 2 to 5. | 5 to 10. | 10 to 15. | 15 to 20. | 20 to 30. | 30 to 40. | 40 to 50. | 50 to 60. | 60 to 70. | 70 to 80. | 80 to 90. | 90 to 100. | 100 to 110. | Total. |
|---|---|---|---|---|---|---|---|---|---|---|---|---|---|---|---|---|---|---|
| Anæmia | | 1 | | | | | 1 | | | | | | | | | | | 1 |
| Angina Pectoris | 1 | 1 | | | | | | | 1 | 1 | | | | | | | | 2 |
| Aneurism of Aorta | 2 | 1 | | | 1 | | | | | 2 | | | | | | | | 3 |
| Disease of Heart | 29 | 22 | 1 | 3 | 3 | 1 | 2 | 4 | 13 | 2 | 5 | 7 | 4 | 4 | 1 | 1 | | 51 |
| Dropsy " | 4 | 5 | | 1 | 1 | 1 | | | 1 | | 1 | | 2 | 2 | | | | 9 |
| Enlargem't. " | 4 | 1 | | 1 | 1 | | | | | 1 | | | 1 | 1 | | | | 5 |
| Gout " | 1 | | | | | | | | | | | 1 | | | | | | 1 |
| Inflammat'n " | 2 | 1 | 1 | | | | | | | 1 | | | | 1 | | | | 3 |
| Ossification " | 1 | | | | | | | | | | 1 | | | | | | | 1 |
| " Arteries | 1 | | | | | | | | | | | | 1 | | | | | 1 |
| | 45 | 32 | 2 | 5 | 6 | 2 | 3 | 4 | 15 | 7 | 7 | 8 | 8 | 8 | 1 | 1 | | 77 |

6. *Digestive Organs.*

| | Male. | Female. | Under 1 yr. | 1 to 2. | 2 to 5. | 5 to 10. | 10 to 15. | 15 to 20. | 20 to 30. | 30 to 40. | 40 to 50. | 50 to 60. | 60 to 70. | 70 to 80. | 80 to 90. | 90 to 100. | 100 to 110. | Total. |
|---|---|---|---|---|---|---|---|---|---|---|---|---|---|---|---|---|---|---|
| Abscess, Abdominal | | 1 | | | | | | | 1 | | | | | | | | | 1 |
| Apthæ | 1 | | 1 | | | | | | | | | | | | | | | 1 |
| Cancer of Bowels | | 1 | | | | | | | | | 1 | | | | | | | 1 |
| " Liver | 1 | 1 | | | | | | | | 1 | 1 | | | | | | | 2 |
| " Stomach | 2 | 1 | | | | | | | | | 1 | 1 | 1 | | | | | 3 |
| " Tongue | 1 | | | | | | | | | | | | 1 | | | | | 1 |
| Cirrhosis of Liver | 3 | | | | | | | | | 1 | 1 | 1 | | | | | | 3 |
| Cancrum Oris | 6 | 1 | | 1 | 5 | 1 | | | | | | | | | | | | 7 |
| Colic | 1 | | | | | | | | | | | | 1 | | | | | 1 |
| Congestion of Bowels | | 1 | | | | | | | | | | | | | 1 | | | 1 |
| Consumption " | 1 | 1 | | | | | | | 1 | 1 | | | | | | | | 2 |
| Disease of Abdomen | | 1 | | | 1 | | | | | | | | | | | | | 1 |
| " Bowels | | 2 | | | | | | 1 | | | | 1 | | | | | | 2 |
| " Liver | 2 | 2 | | | 1 | | | | | 1 | 1 | 1 | | | | | | 4 |
| " Stomach & Bowels | 4 | 2 | 2 | 1 | | | | 1 | 1 | | | | 1 | | | | | 6 |
| Dropsy, Abdominal | 4 | 3 | | | 1 | | | | 1 | 1 | 1 | | 1 | 1 | 1 | | | 7 |
| " of Liver | | 1 | | | | | | | | | | 1 | | | | | | 1 |
| Enlargement of Liver | | 1 | | | | 1 | | | | | | | | | | | | 1 |
| Fatty Degeneration of Liver | | 1 | | | | | | | 1 | | | | | | | | | 1 |
| Hemorrhage of Bowels | 1 | | | | | | | | 1 | | | | | | | | | 1 |
| " Stomach | 1 | | | | | | | | | | | | 1 | | | | | 1 |
| Hernia | | 2 | | | | | | | | | | | 1 | 1 | | | | 2 |
| Induration of Liver | 1 | | | | | | | | | | | | | 1 | | | | 1 |
| Inflammation of Bowels | | 3 | | | | | | 1 | | | | | 1 | 1 | | | | 3 |
| " Liver | 4 | 9 | 1 | | | | | 1 | 3 | 2 | 1 | 2 | 2 | 1 | | | | 13 |
| " Peritoneum | 3 | 16 | 1 | | | | | 1 | 6 | 8 | 2 | | 1 | | | | | 19 |
| " Stom. & Bowels | 11 | 17 | 5 | | 2 | 3 | 3 | 1 | 4 | 2 | 3 | 2 | 1 | 1 | 1 | | | 28 |
| Intussusception of Bowels | 2 | | 2 | | | | | | | | | | | | | | | 2 |
| Jaundice | 9 | 1 | 4 | | 1 | | | | 1 | | 1 | | 1 | 1 | 1 | | | 10 |
| Obstruction of Bowels | 2 | | | | | | | 1 | 1 | | | | | | | | | 2 |
| Schirrus of Stomach | 1 | | | | | | | | | | | | 1 | | | | | 1 |
| Softening " | | 1 | | | | | | | | 1 | | | | | | | | 1 |
| Teething | 1 | 1 | 1 | 1 | | | | | | | | | | | | | | 2 |
| Ulceration of Bowels | | 3 | | 1 | | | 1 | | | | 1 | | | | | | | 3 |
| " Pharynx | 1 | | | 1 | | | | | | | | | | | | | | 1 |
| " Stomach | | 1 | | | | | | | | | | 1 | | | | | | 1 |
| Worms | | 1 | | | | | 1 | | | | | | | | | | | 1 |
| | 63 | 75 | 17 | 5 | 11 | 5 | 5 | 7 | 21 | 18 | 14 | 10 | 14 | 7 | 4 | | | 138 |

7. *Urinary Organs.*

| | Male. | Female. | Under 1 yr. | 1 to 2. | 2 to 5. | 5 to 10. | 10 to 15. | 15 to 20. | 20 to 30. | 30 to 40. | 40 to 50. | 50 to 60. | 60 to 70. | 70 to 80. | 80 to 90. | 90 to 100. | 100 to 110. | Total. |
|---|---|---|---|---|---|---|---|---|---|---|---|---|---|---|---|---|---|---|
| Diabetes | 1 | | | | | | | | 1 | | | | | | | | | 1 |
| Disease of Bladder | 1 | | | | | | | | | | | | 1 | | | | | 1 |
| " Kidneys | 1 | | | | | | | | 1 | | | | | | | | | 1 |
| Dysuria | 1 | | | | | | | | | | | | | | 1 | | | 1 |
| Inflammation Bladder | 1 | | | | | | | | | 1 | | | | | | | | 1 |
| " Kidneys | | 2 | | | | | | | | | 1 | | | | 1 | | | 2 |
| | 5 | 2 | | | | | | | 2 | 1 | 1 | | 1 | | 2 | | | 7 |

8. *Organs of Generation.*

| | Male. | Female. | Under 1 yr. | 1 to 2. | 2 to 5. | 5 to 10. | 10 to 15. | 15 to 20. | 20 to 30. | 30 to 40. | 40 to 50. | 50 to 60. | 60 to 70. | 70 to 80. | 80 to 90. | 90 to 100. | 100 to 110. | Total. |
|---|---|---|---|---|---|---|---|---|---|---|---|---|---|---|---|---|---|---|
| Cancer of Uterus | | 7 | | | | | | | | 1 | 1 | 2 | 1 | 2 | | | | 7 |
| Child-bed, | | 2 | | | | | | | 1 | 1 | | | | | | | | 2 |
| Convulsions, puerperal | | 8 | | | | | | 1 | 2 | 2 | 3 | | | | | | | 8 |
| Disease of Ovaries | | 3 | | | | | | | | | 2 | | | | 1 | | | 3 |
| " Uterus | | 1 | | | | | | | | | | 1 | | | | | | 1 |
| Gangrene " | | 1 | | 1 | | | | | | | | | | | | | | 1 |
| Hemorrhage " | | 3 | | | | | | | 2 | 1 | | | | | | | | 3 |
| Inflam. " | | 2 | | | | | | | | 1 | | | | 1 | | | | 2 |
| Ulceration " | | 1 | | | | | | | | 1 | | | | | | | | 1 |
| | | 28 | | 1 | | | | 1 | 5 | 7 | 6 | 3 | 1 | 3 | 1 | | | 28 |

9. *Organs of Locomotion.*

| | Male. | Female. | Under 1 yr. | 1 to 2. | 2 to 5. | 5 to 10. | 10 to 15. | 15 to 20. | 20 to 30. | 30 to 40. | 40 to 50. | 50 to 60. | 60 to 70. | 70 to 80. | 80 to 90. | 90 to 100. | 100 to 110. | Total. |
|---|---|---|---|---|---|---|---|---|---|---|---|---|---|---|---|---|---|---|
| Caries of Spine | | 1 | | | | | | | | | | 1 | | | | | | 1 |
| Disease " | 1 | 3 | 2 | | 1 | | | | | | 1 | | | | | | | 4 |
| Inflammation of Hip Joint | | 2 | | | 1 | | 1 | | | | | | | | | | | 2 |
| Rheumatism | 1 | | | | | | | | | | | | 1 | | | | | 1 |
| Spina Bifida | | 2 | 2 | | | | | | | | | | | | | | | 2 |
| | 2 | 8 | 4 | | 2 | | 1 | | | | 1 | 1 | 1 | | | | | 10 |

10. *Integumentary System.*

| | Male. | Female. | Under 1 yr. | 1 to 2. | 2 to 5. | 5 to 10. | 10 to 15. | 15 to 20. | 20 to 30. | 30 to 40. | 40 to 50. | 50 to 60. | 60 to 70. | 70 to 80. | 80 to 90. | 90 to 100. | 100 to 110. | Total. |
|---|---|---|---|---|---|---|---|---|---|---|---|---|---|---|---|---|---|---|
| Ichthyosis | 1 | | 1 | | | | | | | | | | | | | | | 1 |
| Purpura Hemorrhagica | | 2 | | | | | 2 | | | | | | | | | | | 2 |
| | 1 | 2 | 1 | | | | 2 | | | | | | | | | | | 3 |

11. *Old Age.*

| | Male. | Female. | Under 1 yr. | 1 to 2. | 2 to 5. | 5 to 10. | 10 to 15. | 15 to 20. | 20 to 30. | 30 to 40. | 40 to 50. | 50 to 60. | 60 to 70. | 70 to 80. | 80 to 90. | 90 to 100. | 100 to 110. | Total. |
|---|---|---|---|---|---|---|---|---|---|---|---|---|---|---|---|---|---|---|
| Old Age | 25 | 30 | | | | | | | | | | | 10 | 17 | 20 | 8 | | 55 |

12. *External Causes.*

| | Male. | Female. | Under 1 yr. | 1 to 2. | 2 to 5. | 5 to 10. | 10 to 15. | 15 to 20. | 20 to 30. | 30 to 40. | 40 to 50. | 50 to 60. | 60 to 70. | 70 to 80. | 80 to 90. | 90 to 100. | 100 to 110. | Total. |
|---|---|---|---|---|---|---|---|---|---|---|---|---|---|---|---|---|---|---|
| Asphyxia | 6 | 2 | 8 | | | | | | | | | | | | | | | 8 |
| Burns | 9 | 11 | | 6 | 5 | 3 | | | 5 | | 1 | | | | | | | 20 |
| Casualties | 24 | 7 | 2 | | 1 | 1 | | 1 | 12 | 3 | 3 | 3 | 2 | 2 | 1 | | | 31 |
| Drowned | 11 | 3 | | | 1 | 1 | 2 | | 2 | 5 | 3 | | | | | | | 14 |
| Exhaustion | 1 | | 1 | | | | | | | | | | | | | | | 1 |
| Exposure | 2 | 1 | | | | | | | 1 | | | | | 2 | | | | 3 |
| Fracture | 2 | | | | | | | 1 | | | | | 1 | | | | | 2 |
| " Skull | 2 | 1 | | | 1 | | | | 1 | | | | 1 | | | | | 3 |
| Intemperance | 3 | 1 | | | | | | | | | 3 | 1 | | | | | | 4 |
| Neglect | | 2 | | 2 | | | | | | | | | | | | | | 2 |
| Strangulation | 1 | | | | | | | | | | 1 | | | | | | | 1 |
| Suicide | 7 | 1 | | | | | | | 3 | 2 | 1 | 2 | | | | | | 8 |
| Suffocation | 1 | 3 | 2 | | | | | | | 2 | | | | | | | | 4 |
| Wound | 1 | | | | | | | | 1 | | | | | | | | | 1 |
| | 70 | 32 | 13 | 8 | 8 | 5 | 2 | 2 | 25 | 12 | 12 | 6 | 4 | 4 | 1 | | | 102 |
| Still Born | 77 | 55 | 132 | | | | | | | | | | | | | | | 132 |
| Unknown | 34 | 23 | 26 | 4 | 1 | | 1 | 1 | 6 | 6 | 1 | 9 | 2 | | | | | 57 |

TABLE NO. 3.

*Deaths for the First Quarter of* 1855, *at fifteen distinct periods of life.*

| | |
|---|---|
| Under 1 year, . . . . . . | 555 |
| 1 to 2 . . . . . . . | 249 |
| 2 to 5 . . . . . . . | 226 |
| 5 to 10 . . . . . . . | 91 |
| 10 to 15 . . . . . . . | 44 |
| 15 to 20 . . . . . . . | 75 |
| 20 to 30 . . . . . . . | 316 |
| 30 to 40 . . . . . . . | 230 |
| 40 to 50 . . . . . . . | 141 |
| 50 to 60 . . . . . . . | 120 |
| 60 to 70 . . . . . . . | 150 |
| 70 to 80 . . . . . . . | 114 |
| 80 to 90 . . . . . . . | 54 |
| 90 to 100 . . . . . . . | 19 |
| 100 to 110 . . . . . . . | 0 |
| | 2394 |
| Still Born . . . . . . . . | 132 |
| Total, | 2526 |

Included within the above table, were 234 from the Blockley Alms House; 140 Blacks, and 18 from the country, as follows:

| | January. | February. | March. | Total. |
|---|---|---|---|---|
| Almshouse, . . . | 72 | 86 | 76 | 234 |
| Blacks, . . . | 45 | 36 | 59 | 140 |
| Country, . . | 5 | 6 | 8 | 19 |
| Total, | | | | 393 |

